# American vs. Foreign Pharmaceutical Articles

**Published:** 1893-05

**Authors:** 


### AMERICAN *VS.* FOREIGN PHARMACEUTICAL ARTICLES.

Inconsistency is probably the most common failing of mankind. We find a lot of doctors who are unwilling to use an American proprietary article, such as advertised in the medical journals, yet doctors and medical journals take up foreign articles, even when the formula is *secret* and *patented,* and use them.

Why can't an American pharmacist make as good a thing as a German, for instance? Further, the former doesn't talk about a trademark and conceal his formula.

This country is getting old enough to let go the leading strings and lead itself. If you can get a good non-secret preparation in America what on earth is the use of importing some patented article from abroad? Be consistent.

